# Anterior Spinal Artery Syndrome in a Patient With Multilevel Cervical Disc Disease: A Case Report

**DOI:** 10.7759/cureus.64577

**Published:** 2024-07-15

**Authors:** Faisal Althobaiti, Rayan Maghrabi, Naif Alharbi, Mohammed Alwadai, Maha K Almatrafi, Somaya Bajammal

**Affiliations:** 1 Neurology, King Fahad General Hospital, Jeddah, SAU; 2 Medicine and Surgery, Umm Al-Qura University, Makkah, SAU

**Keywords:** fibrocartilaginous embolism, cervical disc, paraplegia, vascular myelopathy, spinal stroke, anterior spinal cord infarction

## Abstract

Anterior spinal artery syndrome (ASAS) is a rare form of spinal cord infarction, making its incidence and prevalence difficult to determine. We present the case of a 60-year-old woman with multiple vascular risk factors who experienced a sudden onset of severe lower limb weakness, raising immediate concerns about spinal cord ischemia. Diagnostic evaluations confirmed ASAS, although the exact cause and mechanism of her spinal cord infarction remained undetermined. The potential presence of significant cervical disc disease suggests fibrocartilaginous embolism (FCE) as a possible underlying mechanism, despite the lack of direct evidence. This case underscores the importance of clinical awareness and timely intervention in patients with similar symptoms and vascular risk factors. Early recognition, cause identification, and appropriate management are crucial for improving outcomes in spinal cord ischemia, guiding specific treatment strategies, and potentially preventing recurrence.

## Introduction

Anterior spinal artery syndrome (ASAS) is a subtype of spinal cord infarction. The incidence and prevalence of spinal cord infarction are not well established due to its rarity [1]. Nonetheless, one study has indicated that it accounts for only 1%-2% of all vascular neurological pathologies [1]. ASAS typically presents with sudden back pain, followed by the development of bilateral paralysis, most commonly affecting the lower limbs. It is characterized by areflexia and a loss of pain and temperature sensation, while proprioception and vibration sense are usually spared. Autonomic dysfunction, including bladder, bowel, and sexual dysfunction, has also been reported as a manifestation of ASAS [2]. The most common cause of ASAS is aortic disease, such as aortic dissection, aortic aneurysms, and vasculitis, or iatrogenic factors, including surgeries or procedures that result in direct trauma to the aorta or intraoperative hypotension, or embolization. Other reported causes include disc herniation, kyphoscoliosis, atlanto-occipital dislocation, spinal cord neoplasia, sickle cell disease, polycythemia, and infections like syphilis, schistosomiasis, tuberculosis, and Neisseria meningitidis [3].

Here, we present a case of ASAS in a 60-year-old woman with notable cervical disc disease. There is a likelihood of fibrocartilaginous embolism (FCE) as a potential underlying mechanism. 

## Case presentation

A 60-year-old Saudi woman with a history of diabetes mellitus (DM) type 2, hypertension, and knee osteoarthritis presented to the emergency room with a sudden headache, abdominal pain, and lower limb weakness lasting one day. She reported being in her usual state of health until she experienced a sudden onset of severe upper abdominal pain that was localized and sharp. The pain did not radiate and had no relieving or aggravating factors. This was followed by a sudden loss of function in her lower limbs bilaterally; she could neither walk nor move them against gravity. Additionally, she was unable to pass urine and complained of fecal incontinence. The patient admitted to experiencing similar symptoms about five months prior which resolved spontaneously without seeking medical attention. She denied any other complaints including neck or back pain as well as any history of hypotensive episodes, trauma, or recent surgeries. 

On presentation, she was conscious, alert, and oriented to time, person, and place. Her vital signs were stable with a blood pressure reading of 120/70 mmHg, heart rate of 74 bpm, respiratory rate of 20 breaths per minute, and temperature measured at 36.7 degrees Celsius.

Neurological examination has revealed intact cranial nerves. Normal upper limb tone, power 4/5, and +1 reflexes with intact sensation. For the lower limb exam, there was bilateral symmetrical flaccid paralysis, power 0/5, areflexia, and sensory level up to T4-T6, with preserved proprioception sense. She also had a loss of anal tone and urinary retention. 

**Figure 1:**
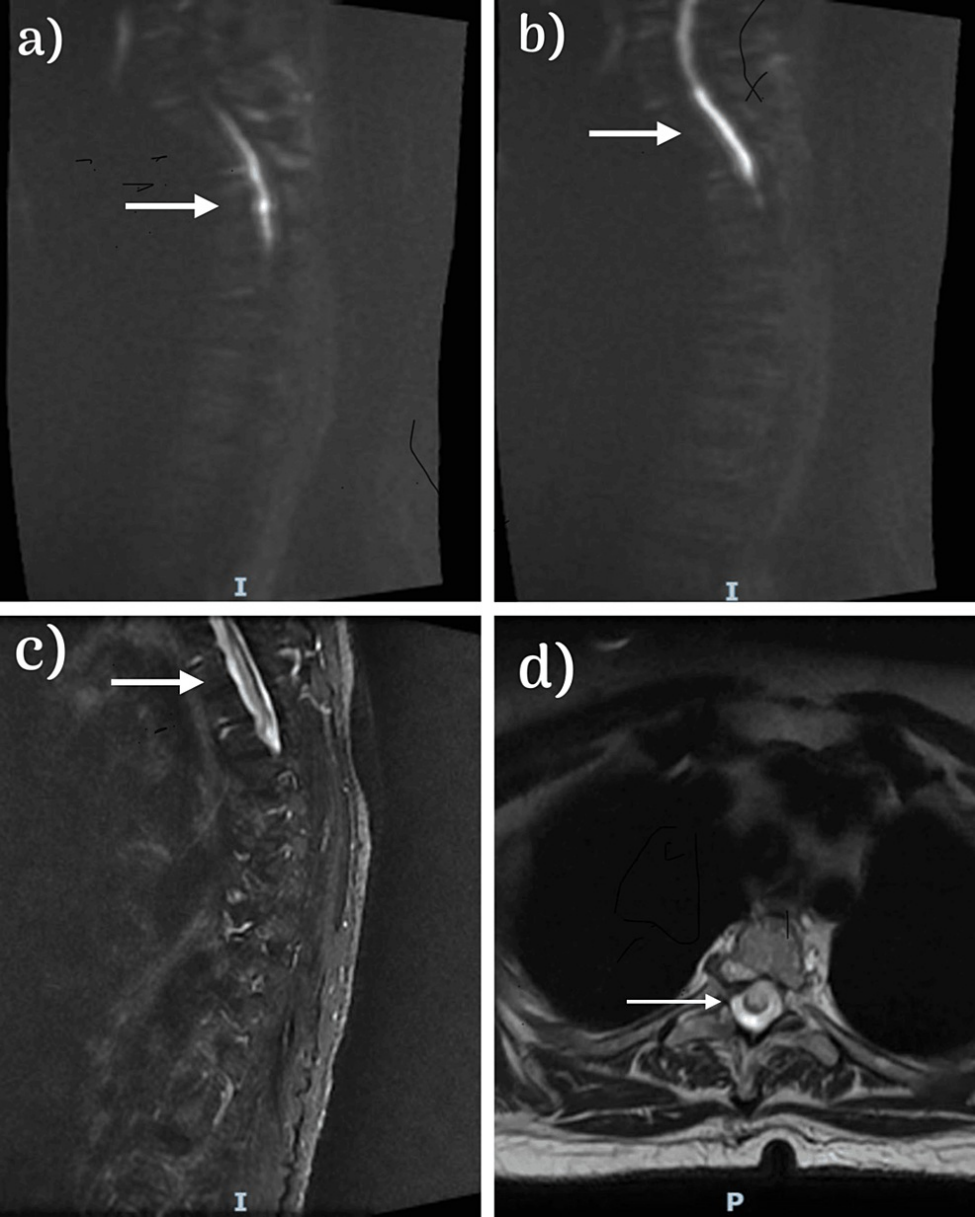
Magnetic resonance imaging of the cervical and thoracic spines (a, b) Restricted diffusion on the diffusion-weighted imaging in the spine from T1 to T7. (c, d) A long segment of intramedullary hyperintensity observed in the T2 image. This hyperintensity is mainly located ventrally and to a lesser extent centrally at the level T1-T7.

**Figure 2:**
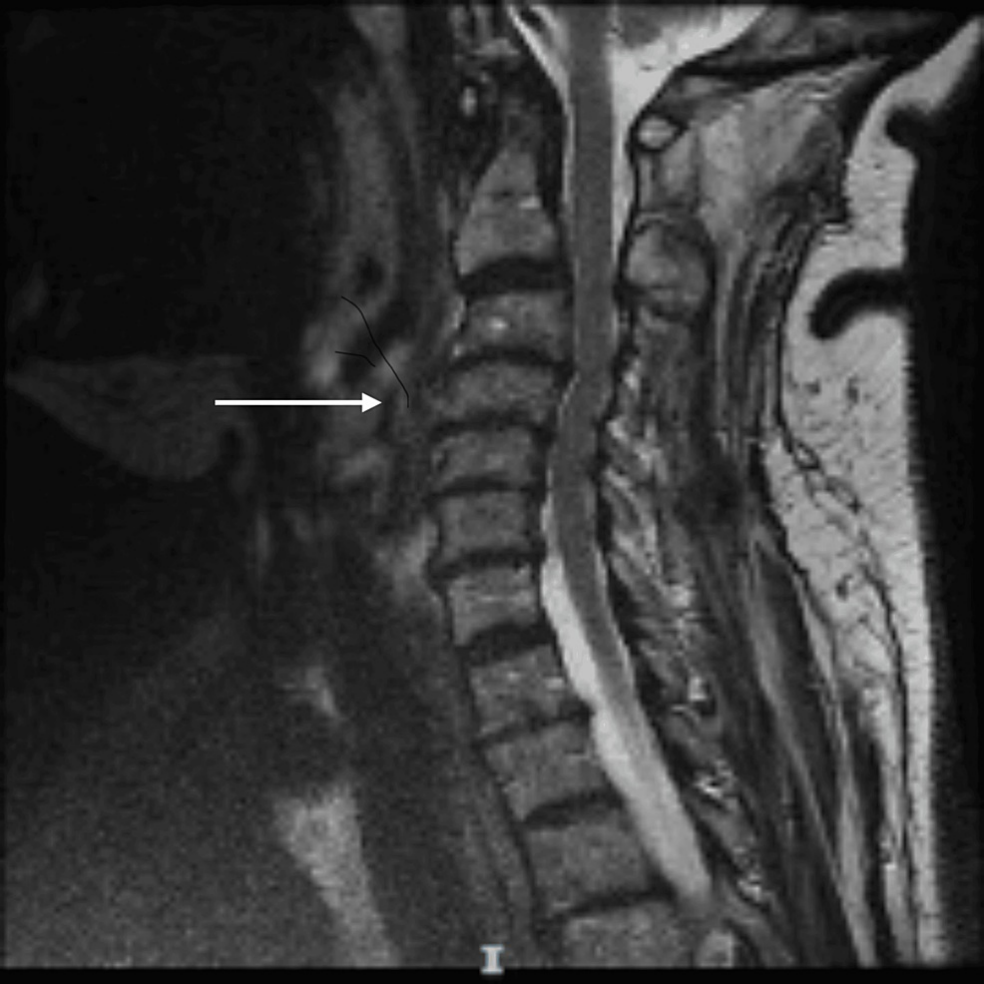
Magnetic resonance imaging of the cervical spine T2-weighted image of the cervical spine depicting multilevel mild disc degeneration with C3/4, C4/5, C5/6, and C6/7-disc osteophyte complexes. These findings are associated with evidence of multilevel spinal canal stenosis.

MRI spine with contrast revealed findings of upper thoracic myelopathy suggestive of spinal cord infraction with diffuse restriction and no enhancement (Figures 1a-1d). Significant cervical disc disease with multilevel cervical disc osseous lesions with variable degrees of foraminal and significant central canal compromise was noted (Figure 2). 

**Figure 3:**
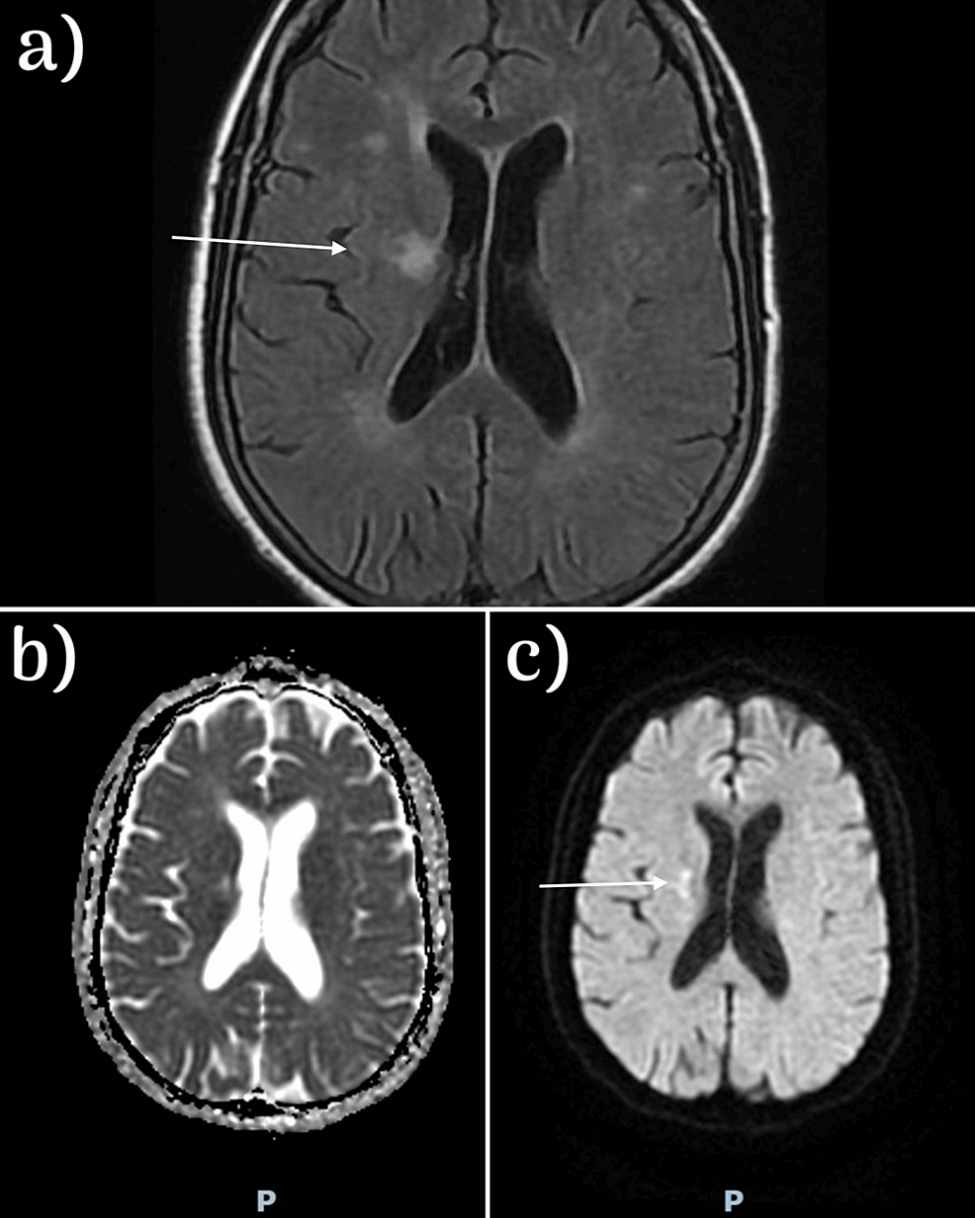
Magnetic resonance imaging of the brain (a) Multiple small scattered areas with high signal intensity observed in the bilateral corona radiata, as well as in the right basal ganglia and periventricular regions on both sides in the T2 FLAIR sequence. (b) No abnormality detected on the apparent diffusion coefficient (ADC) image. (c) A high signal intensity specifically in the right basal ganglia on the diffusion-weighted image (DWI).

CT aortic angiography and CT angiography brain showed no evidence of arterial occlusion, thrombosis, or aneurysmal dilatation. MRI of the brain showed multiple old lacunar infarcts mainly in the corona radiata predominantly on the right side (Figures 3a-3c). Her laboratory workup including complete blood count, chemistry, and coagulation profile were all within normal limits (Table 1). Autoimmune workup and tumor markers were unremarkable (Table 2).

**Table 1:** The laboratory findings include cell blood count, coagulation, and chemistry

Test	Result	Reference range
Complete blood count		
White blood cells	7.89×10^9^/L	4-10
Red blood cells	4.51×10^12^/L	3.8-4.8
Hemoglobin	13.9 g/dL	12-15
Hematocrit	37.3%	36-46
Mean corpuscular volume	85.7 fL	83-101
Mean corpuscular hemoglobin	28.4 pg	27-32
Platelets	226×10^9^/L	150-410
Partial thromboplastin time	29.9	26-40
International normalized ratio	1.1	0.9-1.2
Chemistry		
Creatinine	0.73 mg/dL	0.6-1.2
Blood urea nitrogen	26.4 mg/dL	6-20
Sodium	138 mEq/L	136-145
Chloride	101 mEq/L	96-107
Potassium	3.93 mEq/L	3.5-5.0
Calcium	8.1 mg/dL	8.5-10.3
Magnesium	1.77 mg/dL	1.7-2.4
Low-density lipoproteins	80 mg/dL	<100
Cholesterol	180 mg/dL	<200
Triglyceride	135 mg/dL	<150
Hemoglobin A1C	7%	<6.4%

**Table 2:** The laboratory findings include autoimmune and tumor markers

Test	Result	Reference range
Autoimmune panel		
B-2 Glycoprotein 1 Ab-igg	Negative	< 20 SGU
B-2 Glycoprotein 1 Ab-igm	Negative	<20 SGU
Cardiolipin igg Ab	Negative	<14 GPL
Cardiolipin igm Ab	Negative	<12 MPL
Double-stranded DNA Ab	Negative	<4 IU/mL
Antinuclear Ab	Negative	< 1:80
Rheumatoid factor	9 IU/mL	0-15
C-reactive protein	15.8 mg/L	0-3
Erythrocyte sedimentation rate	19 mm/hr	<20
Tumor markers		
Ca 125	Normal	<35 units per mL
Ca 19-9	22.2 µ/mL	0-37
Ca 15-3	17.3 µ/mL	0-30
Carcinoembryonic antigen	2.31 ng/mL	0-5.5
Alpha-feto protein	4.9 ng/mL	0-10

The echocardiogram revealed hypertensive heart disease with a good systolic function of 58%. The prolonged Holter study was unremarkable and showed no abnormality. We initiated treatment with a single antiplatelet agent (Aspirin 80 mg, once daily) and statin as well as maintained her usual diabetic and hypertension medications. A Foley catheter was inserted for the patient's urinary retention.

After completing all investigations, we have been unable to identify the cause of the spinal cord infarction in this patient. She has been discharged from our facility on aspirin and statin and will now be receiving physiotherapy at an advanced rehabilitation center. In addition, we have arranged for bladder training and geriatric care and advised her to manage her medical background (DM and hypertension) with her family doctor.

After one month of follow-up, the patient's condition has remained unchanged since discharge. She is still wheelchair-bound and continues to experience bilateral lower-limb paralysis. She remains under the care of a rehabilitation center where she is receiving ongoing physiotherapy and bladder training. Regular follow-up appointments have been scheduled to monitor her progress and manage her underlying conditions.

## Discussion

In this report, we present a case of ASAS with an undetermined cause potentially related to FCE. The anterior spinal artery (ASA) arises from the fusion of two branches of the intracranial vertebral arteries at the level of the foramen magnum. Descending along the spinal cords' anterior median sulcus, it supplies blood to the ventral medulla and the anterior two-thirds of the spinal cord. ASAS, alternatively termed anterior cord syndrome, manifests when the ASA is obstructed, impacting the front two-thirds of the spinal cord and leading to neurological complications [4-6]. The abrupt onset of severe weakness in our patient's bilateral lower limbs, especially in the presence of vascular risk factors, prompts concern for spinal cord ischemia. ASAS's clinical manifestations, stemming from compromised spinal cord tracts and lower motor neurons, yield a spectrum of symptoms, posing challenges in management. These symptoms, ranging from neuropathic pain to autonomic dysfunction and severe bowel, bladder, and sexual impairments, underscore the complexity of the condition [6].

Diagnostic imaging is crucial for diagnosing spinal cord infarction. Our patient underwent an MRI of the spine with contrast, which revealed upper thoracic myelopathy indicative of spinal cord infarction, characterized by diffuse restriction and no enhancement. While diffusion imaging revolutionized the diagnosis of brain ischemia years ago, its application to the spine has only been explored in the past decade. This remains technically challenging due to the need for strong gradients, the small size of the spinal cord, and the presence of flow artifacts. MR imaging is the preferred method for diagnosing spinal ischemia and for differential diagnosis. Adding contrast media can be particularly helpful in the acute stage, as the absence of enhancement at this stage can distinguish ischemia from inflammatory, tumoral, or infectious diseases, as demonstrated in our patient, who showed no enhancement [7-9].

When dealing with spinal cord infarction, identifying the cause of the ischemia is crucial to prevent further episodes. In children, spinal cord ischemia is often due to cardiovascular abnormalities and trauma. In adults, the leading causes include aortic disease (such as atherosclerosis, aortic surgeries, and thoracic aortic aneurysms), as well as a clinical history of vascular disease [1,10]. Known vascular risk factors, including hypertension, smoking, hyperlipidemia, and diabetes mellitus, also contribute. Other conditions that can lead to spinal cord ischemia include adjacent spinal degenerative disease, epidural or spinal anesthesia, FCE from disc herniation, vertebral artery dissection, sympathectomy, systemic hypotension, cardiac embolism, coagulopathies, and vasculitis disorders [11-13]. Theories propose that in degenerated discs, the fibrocartilaginous material from the nucleus pulposus has the potential to migrate backward through adjacent blood vessels and ultimately reach the arterial blood supply [14,15]. Interestingly, our patient underwent a comprehensive stroke workup to determine the most likely cause. She did not have common risk factors such as spinal or aortic surgery or episodes of hypotension. However, she did have significant cervical disc disease with multilevel cervical disc osseous lesions, leading to varying degrees of foraminal and significant central canal compromise. Although disc herniation is common, spinal cord infarction resulting from it is rare. Only a few reported cases have indicated a similar potential mechanism [16].

Immediate treatment of the underlying cause of ASAS is crucial to prevent long-term neurological complications. Despite appropriate interventions, full neurological recovery is often unattainable. In a review of 20 childhood cases, only seven children were mobile, all needing assistance to walk. Among 44 adult patients, follow-up of varying durations showed that only 11% walked independently, with one-third requiring support. Therefore, intensive rehabilitation is essential. Our patient has been discharged and will now receive physiotherapy at an advanced rehabilitation center. Additionally, we have arranged for bladder training and nursing care. Successful rehabilitation relies on interdisciplinary support and a patient-centered approach. The surgical and intensivist team transitions care to a physiatry-led rehabilitation team, including neurology, psychiatry, physical and occupational therapy, and at-home care providers. Effective collaboration and communication among these professionals are crucial to meeting the patients' needs [17-19].

## Conclusions

In this report, we present a case of ASAS with multiple cervical disc disease. Despite thorough investigation, we were unable to identify specific factors or underlying conditions contributing to the development of ASAS in this location. The possibility of FCE as a potential underlying mechanism remains speculative without direct evidence. This case highlights the need for further research to better understand the diverse presentations and potential causes of ASAS.
